# GLUT1-DS Italian registry: past, present, and future: a useful tool for rare disorders

**DOI:** 10.1186/s13023-023-02628-2

**Published:** 2023-03-21

**Authors:** Costanza Varesio, Valentina De Giorgis, Pierangelo Veggiotti, Nardo Nardocci, Tiziana Granata, Francesca Ragona, Ludovica Pasca, Martina Maria Mensi, Renato Borgatti, Sara Olivotto, Roberto Previtali, Antonella Riva, Maria Margherita Mancardi, Pasquale Striano, Mara Cavallin, Renzo Guerrini, Francesca Felicia Operto, Alice Pizzolato, Ruggero Di Maulo, Fabiola Martino, Andrea Lodi, Carla Marini

**Affiliations:** 1 Department of Child Neurology and Psychiatry, IRCCS Mondino Foundation (Member of ERN-Epicare), Pavia, Italy; 2 Department of Brain and Behavioral Sciences, University of Pavia, Pavia, Italy; 3 Pediatric Neurology Unit, Vittore Buzzi Hospital, Milan, Italy; 4 Department of Biomedical and Clinical Sciences, Luigi Sacco Hospital, University of Milan, Milan, Italy; 5 Department of Pediatric Neuroscience Fondazione, IRCCS Istituto Neurologico Carlo Besta (Member of ERN-Epicare), Milan, Italy; 6 IRCCS Istituto Giannina Gaslini (Member of ERN-Epicare), Genoa, Italy; 7 Department of Neurosciences, Rehabilitation, Ophthalmology, Genetics, Maternal and Child Health, Università Degli Studi di Genova, Genoa, Italy; 8 Child Neuropsychiatry Unit, IRCCS Istituto Giannina Gaslini (Member of ERN-Epicare), Genoa, Italy; 9 Neuroscience Department, Meyer Children’s University Hospital (Member of ERN-Epicare), Florence, Italy; 10 Child Neuropsychiatry Unit, Department of Medicine, Surgery and Dentistry, University of Salerno, Salerno, Italy; 11 Cloud R S.R.L., Milan, Italy; 12 Associazione Italiana Glut1 aps, Milan, Italy; 13 Child Neurology and Psychiatric Unit, Salesi Children’s Hospital, Ancona, Italy

**Keywords:** GLUT1 deficiency syndrome, Rare disease, Patient registry

## Abstract

**Background:**

GLUT1 deficiency syndrome is a rare, genetically determined neurological disorder for which Ketogenic Dietary Treatment represents the gold standard and lifelong treatment. Patient registries are powerful tools providing insights and real-world data on rare diseases.

**Objective:**

To describe the implementation of a national web-based registry for GLUT1-DS.

**Methods:**

This is a retrospective and prospective, multicenter, observational registry developed in collaboration with the Italian GLUT1-DS association and based on an innovative, flexible and configurable cloud computing technology platform, structured according to the most rigorous requirements for the management of patient’s sensitive data. The Glut1 Registry collects baseline and follow-up data on the patient’s demographics, history, symptoms, genotype, clinical, and instrumental evaluations and therapies.

**Results:**

Five Centers in Italy joined the registry, and two more Centers are currently joining. In the first two years of running, data from 67 patients (40 females and 27 males) have been collected. Age at symptom onset was within the first year of life in most (40, 60%) patients. The diagnosis was formulated in infancy in almost half of the cases (34, 51%). Symptoms at onset were mainly paroxysmal (mostly epileptic seizure and paroxysmal ocular movement disorder) or mixed paroxysmal and fixed symptoms (mostly psychomotor delay). Most patients (53, 79%) are currently under Ketogenic dietary treatments.

**Conclusions:**

We describe the principles behind the design, development, and deployment of the web-based nationwide GLUT1-DS registry. It represents a stepping stone towards a more comprehensive understanding of the disease from onset to adulthood. It also represents a virtuous model from a technical, legal, and organizational point of view, thus representing a possible paradigmatic example for other rare disease registry implementation.

**Supplementary Information:**

The online version contains supplementary material available at 10.1186/s13023-023-02628-2.

## Background

Glucose transporter type 1 deficiency syndrome (GLUT1-DS) is a rare, genetically determined, treatable neurological disorder with an incidence of 1.65–2.22 per 100,000 births. In retrospective studies, the prevalence of GLUT1-DS was estimated to be around 1:90,000 [1, 2]. Yet, considering mild phenotypes that might remain undiagnosed the actual prevalence of the GLUT1-DS spectrum might be underestimated [3]. A broad spectrum of manifestations with variable degrees of severity and different timing of presentation throughout a patient’s life is described. Common features are represented by a combination of drug-resistant often generalized epilepsy, microcephaly, cognitive and motor impairment, and persistent or paroxysmal movement disorders [1, 4–6]. Symptoms are often combined or subsequent and a variable degree of cognitive and motor impairment emerges in most but not all patients.

GLUT1-DS is caused by pathogenic variants in the *SCL2A1* gene, haploinsufficiency leads to decreased availability of glucose in the brain thus impacting brain function [1, 4]. Diagnosis of GLUT1-DS is supported by hypoglycorrhachia (reduced glucose concentration in cerebrospinal fluid (CSF)) in a setting of normoglycemia [1].

Classic Ketogenic Diet (cKD) is effective in reducing seizures, and movement disorders [7–11]. Triheptanoin was recently administered, in a randomized double-blind trial, in GLUT1-DS patients not on cKD to evaluate the effect on epileptic seizure [12]. Although GLUT1-DS has gained the attention of many researchers and has been the target of several clinical and genetic studies, there is still poor knowledge on some aspects including epidemiology, natural history, risk of comorbidities, response to KDTs, long-term consequences, and mortality.

A comprehensive understanding of the natural course of the disease is essential and requires examining a large sample over a prolonged period. Disease and patient registries are vital tools to collect, store, and manage clinical and genetic data that can be used to monitor disease and improve diagnostic and management practices. Patient rare disease registries are crucial to shed light on natural history and long-term outcome with or without treatment. In addition, they contribute to increasing opportunities for coordinating healthcare expertise, facilitating research and increasing access to clinical trials of new therapies [13–16]. To improve awareness and understanding of the disease and accelerate research into GLUT1-DS, the Italian GLUT1-DS Association aimed to create a National Patient Registry. The large-scale observational data collected over an extended period included in the registry will be essential to define clinical characteristics and outcomes of GLUT1-DS and to provide further evidence for adequate clinical, instrumental and therapeutic management and surveillance.

Herein, we describe the design, development, and deployment of a national web-based, user-friendly, and interoperable registry for GLUT1-DS to highlight the valuable nature of this tool. We also illustrate the potential for expansion to other possible partners worldwide.

## Methods

### Study design

The Italian GLUT1-DS registry was developed, starting in 2018, under the auspices of the Italian GLUT1-DS Association to bring together a single registry, of subjects with GLUT1-DS from expert centers disseminated on the National Territory.

The study was designed as a multicenter, observational, retrospective and prospective, web-based, cloud-based, rare disease registry and conducted under conditions of routine clinical practice, according to the treating clinicians.

Based on the Cloud-R RD^®^ platform (https://www.cloud-r.eu/en/patient-registry), the GLUT-DS Registry customization was designed using a co-design approach that saw the participation, in addition to the Information Technology (IT) experts, of Steering Committee Clinicians and Patients’ Representatives.

The AGILE project (https://www.agilealliance.org/) management method was applied. The design was developed with Agile Methodology with the representatives of users (clinicians and patients) throughout different incremental releases of the Registry prototype until the final version was validated by all representatives of users and entered into production.

Customization is guaranteed by the presence of a core software that does not have to be modified each time, but on which the necessary interfaces and datasets and connections are inserted. To allow this approach, the Cloud-R RD^®^ platform has been designed and built to be solid, secure, and scalable; it applies the highest IT security standards and a privacy by design approach. Furthermore, the platform is multilingual by design, allowing the rapid implementation of services at an international level.

### Registry governance

The Italian GLUT1DS Association is the owner of the registry and its coordination is entrusted by a Scientific Advisory Board (represented by GLUT1-DS expert clinicians and patients’ representatives). The registry Steering Committee defined the dataset and the protocol for data collection and analysis, the characteristics of the GLUT1-DS Registry customization, the policy, and the rules for the use of data, the project’s budget, and its long-term sustainability. The Registry Steering Committee establishes and manages relationships with regulators, researchers, and pharmaceutical companies that will be instrumental in fostering research.

An external Advisor on rare diseases (Kaleidos social cooperative society—https://www.kaleidos.care/english/) was appointed to manage legal fulfillments.

Furthermore, a registry manager, specifically trained in pediatric neurology, was appointed to support the activities of the Steering Committee and of the national centers participating in the project in entering data and guaranteeing uniformity and completeness of collected data.

The IT infrastructure and the entire application service are provided and managed by Cloud-R s.r.l (https://www.cloud-r.eu/en) thanks to its Cloud-R RD^®^ platform and its “Software as a Service” (SaaS) service model.

The administrative and financial support is guaranteed by the Italian GLUT1-DS Association and by an awarded grant from Pierfranco e Luisa Mariani Foundation (https://www.fondazione-mariani.org/) for the years 2019–2020.

Currently, five Italian Centers are involved: Meyer Children Hospital in Florence, Besta Neurological Institute in Milan, Buzzi Children Hospital in Milan, Mondino Neurological Institute in Pavia, and IRCCS ‘G. Gaslini’ Children Hospital, Genoa.

### Recruitment, data collection and data protection

The registry includes individuals with:

- confirmed diagnosis of GLUT1DS based on the combination of characteristic clinical features, hypoglycorrhachia and *SLC2A1 gene* variants
- possible diagnosis in those subjects in whom clinical features were not accompanied by hypoglycorrhachia yet carrying a *SLC2A1 gene* variant.
- uncertain in those individuals with clinical features suggestive of GLUT1DS and with hypoglycorrhachia but no detectable *SLC2A1 gene* variant or deletions.

Exclusion criteria are represented by the presence of suggestive clinical features without hypoglycorrhachia and/or *SLC2A1* pathogenic variants.

Participation in the Registry is voluntary. Patients, parents or legal guardians must provide written informed consent to participate in the Registry. The consent obtained from each participant allows access to medical records and test results, to contact patients over time and to include the data collected in scientific publications.

Participants’ data entry is carried out by the treating clinician or the registry manager. The Registry is designed to capture longitudinal data on GLUT1-DS. The data source is represented by the patient’s medical record. The data are captured in a multimodule system. The clinician user or the Registry Manager user must complete all the mandatory fields to save the entry. The system allows editing and/or completion of previously unanswered non-mandatory fields at the user’s convenience. The complete dataset with details on mandatory and non-mandatory fields is provided in Additional Material 1.

In the co-design phase, a minimum dataset was defined by the Steering Committee based on the information collected and clinical/instrumental evaluations performed in the daily clinical practice in GLUT1 patients, to have a core data set as complete and reliable as possible and to minimize missing data for each patient.

Each enrolled patient’s clinical, genetic, and therapeutic information is recorded retrospectively in specific sections “Patient Data” and “Diagnosis” which include the following dataset: personal data, residence data, contact data, family history, diagnosis and information about onset and the 1st year of the disease. Longitudinal data are captured through a specific “follow-up” section which includes the following monitors: follow-up visits, blood tests, cerebrospinal fluid tests and other instrumental exams, diet therapies and other therapies forms (Table 1; Additional file 1).

**Table 1:** Overview of data collected

Dataset Name	Dataset content
*Patient Data*	
Personal data	Demographic information
*Residence data*	
Contact data	Who to call to share information about the patient
Family history	Consanguinity, family history, other neuropsychiatric symptoms, or symptoms possibly related to GLUT1DS
Consent	Consent to participation in the Register of People with GLUT1 Deficit Syndrome and Date of acquisition of the informed consent
Diagnosis data	Date of diagnosis, genotype, inheritance
Symptoms at onset	Age at onset and first symptoms observed
*Follow-up*	
Follow-up visits	Recording of clinical, auxological, neurological data at each follow-up
Follow-up exams	Recording of all exams performed at each follow-up with related reports
*Therapies*	
Therapies for epileptic seizures	Recording of all pharmacological treatments carried out over time and their efficacy
*Therapies for movement disorder*	
Dietary treatments	Recording of information about ketogenic dietary treatments and their efficacy

The Italian GLUT1-DS registry benefits from an infrastructure characterized by a robust security system. The platform Cloud-R RD^®^ complies with EU Regulation 2016/679. Data is stored in cloud-based servers managed by Cloud-R RD^®^, on certified Google IaaS infrastructure. The data and resources relating to these servers are segregated in geographical areas within the European Union. Users access the system through 2 Factor Authentication, which provides the top level of cybersecurity: every authorized user is assigned a personal username and password. Access is guaranteed after further confirmation via a 5-digit code sent via SMS to the user who has performed the authentication. The platform can be used on a web client.

Each clinician user at each center can access only their center patients’ data. In addition, the registry manager can oversee the data for all centers.

Cloud-R technicians do not access personally identifiable information. Only the dataset structure is an object of technical activity, while personal data is managed exclusively by authorized subjects (treating clinicians and registry manager).

Patients are given the possibility to access and inspect their data, although not being able to make any modifications to enter clinical information.

Local Ethic Committees and Italian Health Authorities may claim for patients’ clinical data contained in the registry and the original medical charts exclusively to verify correctness and integrity of entered data. Within the limits of the Italian laws, aggregated and de-identified data might be shared with third party for scientific or research purposes (i.e. clinicians, researchers, and companies in the bio-pharmaceutical sector) after Steering Committee approval. Each patient is assigned a unique study number, not attributable to personal data, which is automatically generated.

Data are aggregated in an anonymized fashion, without the possibility of identification of individual patients. From the Dashboard, the clinicians obtain predefined statistical graphs relating to the aggregate data of the patients belonging to each center and also statistics at a national level with the aggregate data of all the centers. These statistics are updated in real time.

### Data quality assurance

Data quality is guaranteed by several strategic actions. First, data are gathered from trustworthy sources represented by medical reports and other clinical documentation. Second, both expert clinicians and registry manager capture data, thus ensuring a constant double-check to minimize human error. Data entry is facilitated by the presence of drop-down menus with fixed input possibilities for many variables, wherever possible (for further detail on drop-down menu and free text options see also Additional file 1). There are automatic controls that prevent mistakes in data entry on indicators that have been determined in the design phase of the dataset. Moreover, longitudinal data collection guarantees a continue further validation of collected information.

### Ethics

This study is designed and conducted under the Declaration of Helsinki, International Council for Harmonisation. It meets the necessary requirements for compliance with the General Data Protection Regulation (GDPR) and all other applicable Italian laws and regulations. Each participating site obtained Institutional Ethical Committee approval for the study.

### Statistical analysis

Given the observational nature of the study, no number of participants is fixed. However, based on the number of patients regularly followed at participating centers, about 50 patients were expected to be enrolled in the first two years of the project.

**Fig. 1:**
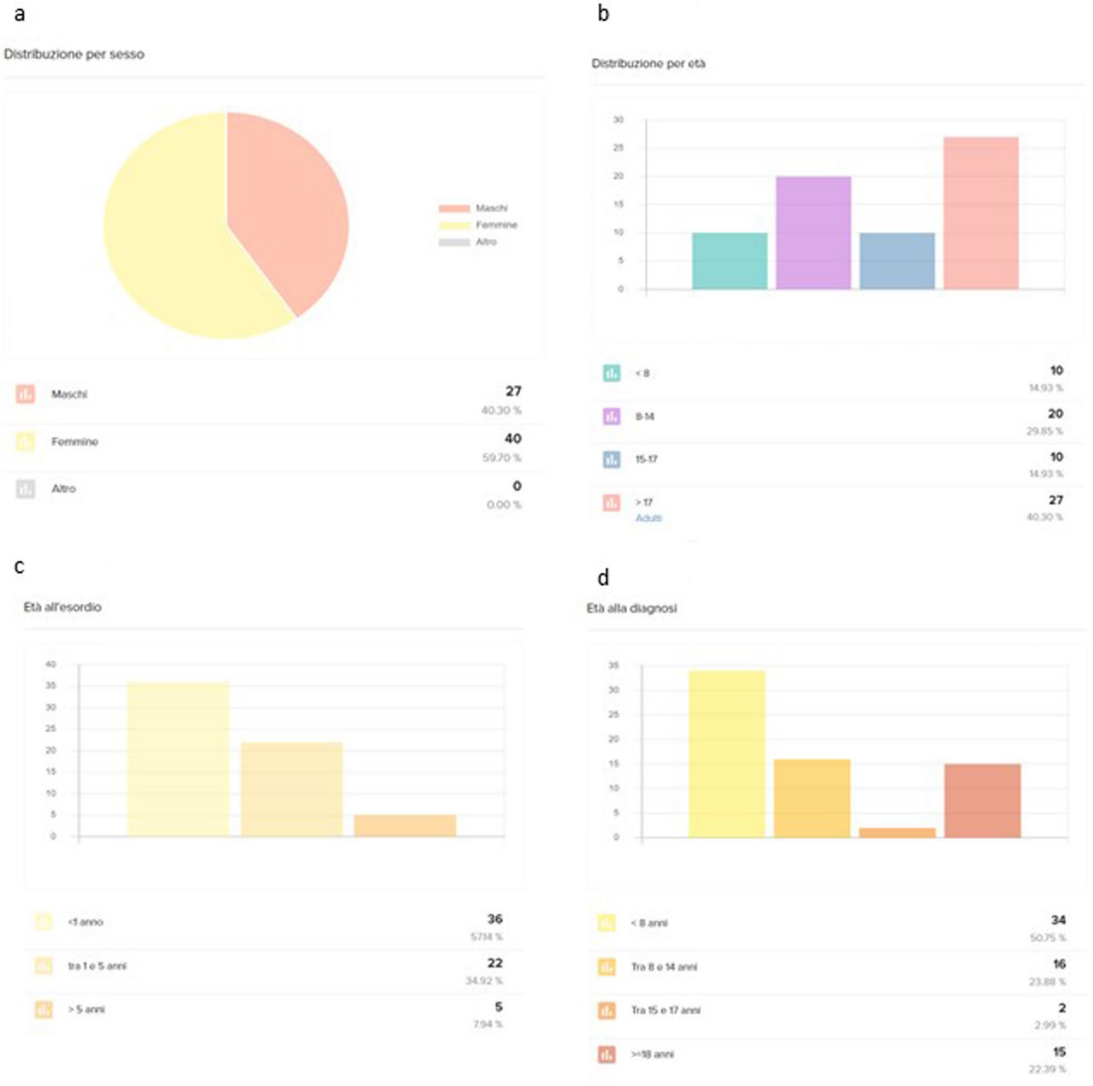
**a**, **b**, **c**, **d** shows examples of constantly updated simple descriptive statistics for demographic variables. **a** sex distribution. **b** age distribution. **c** age at onset. **d** age at diagnosis

Since the registry was developed both as a clinical and research tool, data export, and analysis are crucial. The Cloud-R RD^®^ platform offers constantly updated simple descriptive statistics for selected variables (example provided in Fig. 1).

Users can export data to excel files, under specific permission and rules from the Steering Committee; after evaluation of the request, the activity will be carried out by the provider in compliance with data security/personal data protection regulations and best practices. Technically all levels of data can be exported, but are subject to limitations under data governance rules of the Steering Committee and relevant laws.

## Results

**Fig. 2:**
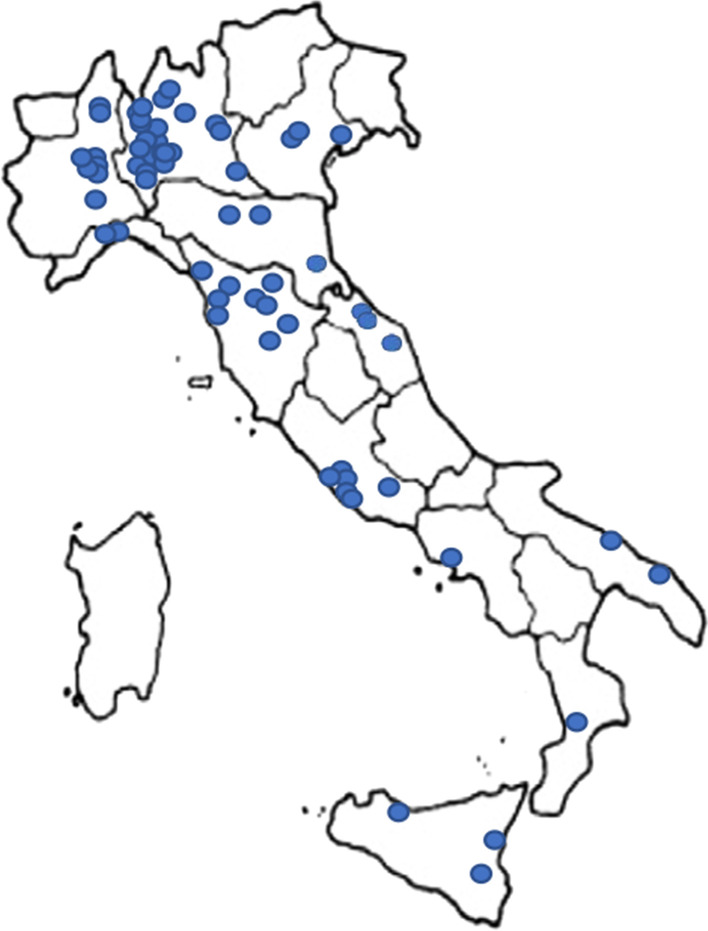
shows geographical distribution of patients participating to the Italian GLUT1-DS Registry

Starting from the 1st of February 2020, 67 individuals were recruited into the registry, and data collection is ongoing. Demographically, the Registry currently consists of 40 females (59.7%) and 27 males (40.3%). Concerning age distribution, we enrolled 12 (17.9%) children (aged < 8 years), 28 (41.8%) pre-adolescent and adolescent (aged between 8 and 17) and 27 (40.3%) adults (Table 1). The geographical origin of entered patients is collected in Fig. 2.

Age at symptoms onset was in the first year of life in 40 patients (60%), between one and five years in 22 patients (33%), and above five years of age in five patients (7%). Age at diagnosis was in infancy in 34 patients (51%), between eight and fourteen years in 16 patients (24%), between 15 and 17 years in 2 patients (3%), and in adulthood in 15 patients (22%) (Table 2). Symptoms at onset were mainly paroxysmal neurologic manifestations (63 patients) or mixed paroxysmal and fixed symptoms (35 patients). Among paroxysmal symptoms, more than half of the patients (56%) had epileptic seizures and paroxysmal ocular movement disorder (20%) (Table 1). The most common of the persistent symptoms was psychomotor delay (70%). Concerning dietary therapies, 53 patients (79%) are currently under Ketogenic dietary treatments, four patients (6%) are treated with triheptanoin, whereas 10 adult patients (15%) are not treated with ketogenic dietary treatments.

**Table 2:** Summary of clinical data of collected patients

Age distribution of pts	gender: F/M	Age at symptoms onset	Age at GLUT1DS diagnosis	Symptoms at onset	ketogenic diet
< 8 years 12 pts (17.9%)	40 females (59.7%)	1st year: 40 patients (60%)	Infancy: 34 pts (51%)	paroxysmal symptoms (63 pts): 56% seizure + paroxysmal ocular movement disorder (20%)	53 patients (79%): KD
8–17 years: 28 pts (41.8%)	27 males (40.3%)	1–5 year: 22 patients (33%)	8–14 years: 16 pts (24%)	mixed paroxysmal and fixed symptoms (35 pts): psychomotor delay (70%)	4 patients (6%): Triheptanoin
> 18 years: 27 pts (40.3%)		> 5 yrs five patients (7%)	15–17 years in 2 pts (3%)		10 patients (15%) not treated
			Adulthood: 15 pts (22%)		

## Discussion

The Italian GLUT1-DS Registry arises from awareness, both of the Association of families and the scientific community, of the need for a national registry assembling clinical, genetic, and follow-up data of a large cohort of subjects with this disorder. To date, incomplete knowledge of the natural history of GLUT1-DS has delayed its complete understanding and the development of the appropriate cure for this disorder. Moreover, GLUT1-DS incidence is likely to be underestimated, and the spectrum of clinical manifestations might be broader than currently known.

In this context, the Italian GLUT1-DS Registry has been designed as an observational retrospective and prospective observational tool to store and retrieve data from routine clinical and therapeutic settings and provide insights into real-world clinical practice. The GLUT1-DS registry aims to collect accurate and homogeneous data on sporadic and familial GLUT1-DS patients, allowing them to draw the longitudinal trajectory and thus, describe the natural, long-term history of the disorder. The registry will have a crucial role in accurately delineating the spectrum of clinical manifestations at disease onset. It will hopefully help identify diagnostic biomarkers, thus ultimately leading to very early recognition and appropriate management of this treatable disorder. In addition, registry data will shed light on the effectiveness and long-term outcome of KDTs. Finally, the registry will also facilitate the identification and recruitment of eligible participants for clinical trials. Hopefully, it will be the starting point for developing new targeted treatments.

The Italian GLUT1-DS registry is the first European registry of the disease. Time elapsed from the Italian GLUT1-DS registry inception and planning in 2018, and its launch and execution in 2020 were relatively rapid. The success of the registry, to date, can be judged by the fact that enrollments foreseen in the first two years of running have been reached.

The registry at present does not have nationwide coverage since participating centers are only distributed in the north and center of Italy. A proposal to join the registry with a provisional acceptance is ongoing for two additional centers in the center and south. However, the patient’s geographical origin is more scattered since families, especially in the south of Italy, tend to move to seek a second opinion.

A previous attempt to conduct a registry on GLUT1-DS was performed in the U.S.A. [17]; however, its conceptualization and management differed substantially from our registry. In particular, in the GLUT1-DS registry developed in the U.S.A. [17], the data were collected and entered directly by families.

High data quality is considered one of the most critical elements in establishing, developing, and long-term maintenance of a rare disease registry [18]. Therefore, the Italian GLUT1-DS registry is built on a robust IT infrastructure, represented by a web-based, user-friendly platform. Other registries in Italy and Europe are based on the Cloud-R RD^®^ platform system: Epidermolysis Bullosa (https://it.eb.rd.cloud-r.eu/) and Poland Syndrome (https://it.poland.rd.cloud-r.eu/) in Italy, hereditary angioedema in France (https://fr.hae.cloud-r.eu/), Greece (https://el.hae.cloud-r.eu/) and Romania (https://ro.hae.cloud-r.eu/). Dynamical configuration capacity, support in technical and organizational design for research groups, co-design Agile support in the definition of the Registry, fast track implementation and lower cost of ownership represent the main advantages of the Cloud-R RD^®^ platform system.

Data quality has been assured by the early appointment of a Registry Manager trained in the field, who can devote time to collecting patients’ medical records and entering data or supervising fairness, adequacy and completeness of clinical data entered by clinicians, thus allowing homogeneous data collection and update on a regular and continuous basis.

The Italian GLUT1-DS registry is currently findable through the Orphanet website (https://www.orpha.net/consor4.01/www/cgi-bin/ResearchTrials_RegistriesMaterials.php?lng=IT&data_id=143875&RegistryMaterialName=Registro-sindrome-da-deficit-di-GLUT1&title=Registro%20sindrome%20da%20deficit%20di%20GLUT1&search=ResearchTrials_RegistriesMaterials_Simple).

A web-based data storage system will guarantee the progressive involvement of other Italian or international centers and subsequent clinical data processing to create a scientific database useful for increasing knowledge of the disease and improving its care, enabling the construction of a collaborative environment at an international level. The platform is natively ready for implementation of interoperability connectors across different standards following specific requirements. Common data elements subsets are possible to be defined with limited effort. Thus, allowing our registry to communicate with other databases and registries, ensuring a certain level of international interoperability. Strong measures were put in place to ensure security and privacy of data collection, maintenance, and use, also thanks to 2 Factor Authentication, which provides the top level of cybersecurity, and to data anonymization.

The processes of patient empowerment in clinical practice and research settings are strictly related to the concepts of patient-centred care and participation in the decision-making process [19]. Our registry is an example of patients organization empowerment in the financial support, and coordination of clinical, research and legal aspects.

For registries to be effective, data input must be complete and comprehensive over long periods. Since GLUT1DS is a chronic disease, which requires constant follow-up of patients over time, we may reasonably assume that data input will be held regularly, with minimal loss to follow-up. The modular design and scalable nature of the registry make it flexible and easily adaptable over time, ensuring its long-term sustainability. Financial sustainability is paramount: maintaining the electronic infrastructure, together with data analysis, interpretation and dissemination are crucial elements for long-term success. The Italian GLUT1DS Association will directly support over time with ad hoc allocated budget costs for the infrastructure maintenance and management activities.

Although outside the primary scope of this paper, we believe some reflections on the preliminary results might be drawn. In line with literature data [1, 20], disease onset was in early childhood in the majority of patients, with epileptic seizure and developmental delay as the first recognized manifestations. Although first symptoms occur in the first year of life in most patients, our preliminary data analysis shows that about one-third of patients were diagnosed in late adolescence or adulthood, thus confirming the observation that diagnostic delay is still a relevant open problem in GLUT1DS.

Our registry is not exempt from some limitations, retrospective data collection poses some issues regarding the completeness of data relating to the early clinical history, especially in patients who received the diagnosis in adulthood. Moreover, some patients may escape, when followed in centers which are not centers of expertise for the disease.

## Conclusions

We describe the principles behind the design, development, and deployment of the web-based nationwide GLUT1-DS registry. The Italian GLUT1-DS registry is an essential clinical and research tool and represents a steppingstone towards a more comprehensive understanding of the disease from the onset on. The Italian GLUT1-DS registry depicts several aspects that make it a virtuous model from a technical, legal, and organizational point of view, thus representing a possible paradigmatic example for other rare disease registry implementations. The added value of the Italian GLUT1-DS registry is the consolidation of the network of interconnected centers across Italy adopting similar diagnostic and treating workflows to stop the migration of patients and their families to a few centers since the best homogenous practice is applied in all national centers. Thanks to its architecture and privacy-by-design approach, the platform enables to manage of patient data (personal, contact, diagnosis, clinical etc.) in compliance with EU Regulation 2016/679, and can be the first step in future development in Real World Data (RWD) directly by patients and for the extension of the registry internationally.

## Supplementary Information

**Additional file 1.** Italian GLUT1-DS Registry dataset

## Data Availability

All data generated or analyzed during this study are included in this published article. Registry protocol will be available upon reasonable request from the corresponding author.

## Abbreviations

cKD: Classic ketogenic diet
CSF: Cerebrospinal fluid
GDPR: General Data Protection Regulation
GLUT1-DS: Glucose transporter type 1 deficiency syndrome
IT: Information technology
KDTs: Ketogenic dietary therapies
RWD: Real world data
SaaS: Software as a service

## References

[1] Klepper J, Akman C, Armeno M, Auvin S, Cervenka M, Cross HJ (2020). Glut1 deficiency syndrome (Glut1DS): state of the art in 2020 and recommendations of the international Glut1DS study group. Epilepsia Open.

[2] Larsen J, Johannesen KM, Ek J, Tang S, Marini C, Blichfeldt S (2015). The role of SLC2A1 mutations in myoclonic astatic epilepsy and absence epilepsy, and the estimated frequency of GLUT1 deficiency syndrome. Epilepsia.

[3] Arsov T, Mullen SA, Damiano JA, Lawrence KM, Huh LL, Nolan M (2012). Early onset absence epilepsy: 1 in 10 cases is caused by GLUT1 deficiency. Epilepsia.

[4] Klepper J, Leiendecker B (2007). GLUT1 deficiency syndrome–2007 update. Dev Med Child Neurol.

[5] Alter AS, Engelstad K, Hinton VJ, Montes J, Pearson TS, Akman CI (2015). Long-term clinical course of Glut1 deficiency syndrome. J Child Neurol.

[6] Pearson TS, Akman C, Hinton VJ, Engelstad K, De Vivo DC (2013). Phenotypic spectrum of glucose transporter type 1 deficiency syndrome (Glut1 DS). Curr Neurol Neurosci Rep.

[7] De Giorgis V, Masnada S, Varesio C, Chiappedi MA, Zanaboni M, Pasca L (2019). Overall cognitive profiles in patients with GLUT1 deficiency syndrome. Brain Behav.

[8] Ito Y, Oguni H, Ito S, Oguni M, Osawa M (2011). A modified Atkins diet is promising as a treatment for glucose transporter type 1 deficiency syndrome. Dev Med Child Neurol.

[9] Kass HR, Winesett SP, Bessone SK, Turner Z, Kossoff EH (2016). Use of dietary therapies amongst patients with GLUT1 deficiency syndrome. Seizure.

[10] Ramm-Pettersen A, Nakken KO, Skogseid IM, Randby H, Skei EB, Bindoff LA (2013). Good outcome in patients with early dietary treatment of GLUT-1 deficiency syndrome: results from a retrospective Norwegian study. Dev Med Child Neurol.

[11] Ramm-Pettersen A, Stabell KE, Nakken KO, Selmer KK (2014). Does ketogenic diet improve cognitive function in patients with GLUT1-DS? A 6- to 17-month follow-up study. Epilepsy Behav EB.

[12] Striano P, Auvin S, Collins A, Horvath R, Scheffer IE, Tzadok M (2022). A randomized, double-blind trial of triheptanoin for drug-resistant epilepsy in glucose transporter 1 deficiency syndrome. Epilepsia.

[13] Ali SR, Bryce J, Kodra Y, Taruscio D, Persani L, Ahmed SF (2021). The quality evaluation of rare disease registries-an assessment of the essential features of a disease registry. Int J Environ Res Public Health.

[14] Boulanger V, Schlemmer M, Rossov S, Seebald A, Gavin P (2020). Establishing patient registries for rare diseases: rationale and challenges. Pharm Med.

[15] Coi A, Santoro M, Villaverde-Hueso A, Di Lipucci Paola M, Gainotti S, Taruscio D (2016). The quality of rare disease registries: evaluation and characterization. Public Health Genom.

[16] Kodra Y, Weinbach J, Posada-de-la-Paz M, Coi A, Lemonnier SL, van Enckevort D (2018). Recommendations for improving the quality of rare disease registries. Int J Environ Res Public Health.

[17] Hao J, Kelly DI, Su J, Pascual JM (2017). Clinical aspects of glucose transporter type 1 deficiency: information from a global registry. JAMA Neurol.

[18] Kodra Y, de la Posada Paz M, Coi A, Santoro M, Bianchi F, Ahmed F (2017). Data quality in rare diseases registries. Adv Exp Med Biol.

[19] Castro EM, Van Regenmortel T, Vanhaecht K, Sermeus W, Van Hecke A (2016). Patient empowerment, patient participation and patient-centeredness in hospital care: a concept analysis based on a literature review. Patient Educ Couns.

[20] De Giorgis V, Veggiotti P (2013). GLUT1 deficiency syndrome 2013: current state of the art. Seizure.

